# Fish-Based Baby Food Concern—From Species Authentication to Exposure Risk Assessment

**DOI:** 10.3390/molecules25173961

**Published:** 2020-08-31

**Authors:** Anna Maria Pappalardo, Chiara Copat, Alessandra Raffa, Luana Rossitto, Alfina Grasso, Maria Fiore, Margherita Ferrante, Venera Ferrito

**Affiliations:** 1 Department of Biological, Geological and Environmental Sciences–Section of Animal Biology “M. La Greca”, University of Catania, 95124 Catania, Italy; alessandra.raffa92@gmail.com (A.R.); lunarossa92@gmail.com (L.R.); 2 Department of Medical, Surgical Sciences and Advanced Technologies “G.F. Ingrassia”, Hygiene and Public Health, University of Catania, I-95123 Catania, Italy; ccopat@unict.it (C.C.); agrasso@unict.it (A.G.); mfiore@unict.it (M.F.); marfer@unict.it (M.F.)

**Keywords:** baby food, DNA barcoding, heavy metals, risk assessment

## Abstract

In this work, two different but complementary approaches were used to evaluate the reliability of fish-based baby foods as a source of safe nourishment for babies. More specifically, barcoding analysis based on the Cytochrome Oxidase I sequences was used for fish species authentication and an analysis of metal/metalloid levels was performed to estimate the exposure risk assessment derived from consumption of selected fish-based baby food in infants and toddlers. COI DNA barcoding revealed that in three samples the species detected did not match the common name of the species shown on the label. In particular, *G. chalcogrammus* and *M. australis* were found in place of *M. merluccius* and *O. mykiss* was found in place of *S. salar*. The analysis of exposure risk assessment indicated a low risk for developing chronic systemic and carcinogenic effects in infants and toddler, under an exposure scenario based on daily consumption of a single box of fish-based baby food. However, it is important to highlight that in order to provide a comprehensive risk assessment it would be important to supplement the levels of exposure resulting from the total diet. Overall, our results suggest that more attention should be paid by authorities to ensure the safety of food for infants and toddlers.

## 1. Introduction

Infants (6–12 months) and toddlers (1–3 years) are vulnerable to dietary exposure to contaminants due to their physiological characteristics, which enhance their vulnerability to the noxious effects of chemicals [1]. For these reasons, the European Union (EU) has issued numerous regulations to guarantee that ready-to-eat foods for infants meet specific requirements for packaging and nutritional, physical, chemical and microbiological safety (EC No 2073/2005, EC No 125/2006, EC No 1881/2006, EC No 141/2006, EU No 10/2011, EC No 52/2016, EC No 213/2018). Concerning the rules for labelling, the regulation EU No 127/2016, which implements regulation EU No 1169/2011, includes the compositional requirements and nutrition declaration for infant formula and follow-on formula ready-for-use. It highlights that labelling, presentation and advertising must provide the necessary information, using terms that do not discourage breast feeding and avoiding any risk of confusion for consumers.

Despite the above regulations, recent literature has focused on the identification and quantification of contaminants in baby food [2,3,4,5]. The products most considered in these studies have been fruit purée, dry baby milk, cereal-based baby foods and meat-based baby food. Fish-based baby foods represent a small fraction of baby food products. However, several investigations have been carried out to establish their toxicant contamination status. For example, levels of PAH markers were higher than the permissible EU limits of 1 mg/kg in 44.4% meat/fish based baby foods in Italy [6]; polychlorinated dibenzo-p-dioxins and dibenzofurans (PCDD/Fs) as well as polychlorinated biphenyls (PCBs) have been measured in samples of commercial fish-based baby food products collected from Spanish markets and pharmacies [7]; mineral oil saturated hydrocarbons (MOSH) contamination was found in fish baby food products [8]; and the contents of minerals, toxic elements and hydroxymethylfurfural (HMF) in infant foods and formulae have been evaluated [9]. While investigations on contamination of fish-based baby food have been common, investigations on fish species authentication are scarce. For example, Quinteiro et al. [10] analyzed samples of baby foods containing hake to evaluate the efficacy of the PCR-RFLP (Polymerase Chain Reaction-Restriction Fragment Length Polymorphisms) methodology for species authentication using the mitochondrial control region as a molecular marker. Fish-based baby food has also been screened using a multiplex polymerase chain reaction (PCR) assay for the rapid identification of ruminant, poultry, fish and pork materials in the framework of preventive measures against the spread of Bovine Spongiform Encephalopathy [11]. Consumer demand for transparency about fish species labeling accuracy has been driven by several factors. Indeed, consumers have become increasingly aware of problems of food fraud and the most common fraud is the substitution of a valuable fish species with a less valuable one. This practice has become very common in the fishing industry due to the overall decrease in the fish catch, the increase in demand for fish from industrialized countries and the globalization of the fish market. The increasing value of fish and seafood products has also greatly increased illegal, unreported and unregulated (IUU) fishing, which has a negative economic impact on the legal commercial fishing industries and fuels commercial fraud [12]. In general, the deliberate or unintentional practice of fish species substitution directly threatens the food security and human health. For example, numerous cases of monospecies fish allergies in children have been reported [13,14,15]; therefore, the substitution of a species with another could expose children under 2 years of age to serious health problems and symptoms ranging from an enterocolitis syndrome to acute urticarial, angioedema or respiratory symptoms [13]. Furthermore, consumers may unknowingly eat a substituted fish species containing a greater quantity of contaminants, such as mercury, than the species declared on the label would contain. This could be of particular concern for pregnant women and young children due to the adverse effects the contaminants can have in particularly sensitive phases of human life. In this respect, intra- and inter-specific variability of metal bioaccumulation (mercury and arsenic) has been statistically assessed in species collected from the north-east and eastern-central Atlantic Ocean in Portuguese waters over one year [16] and different levels of heavy metal bioaccumulation were found in fish species living in the same lagoon off the eastern coast of the Mediterranean Sea [17]. In regard to aquaculture species, significant differences in heavy metal accumulation among three different types of fish culture have been detected in *Sparus aurata* [18] and different levels of contaminants between aquaculture products and wild fish of this species have also been found [19,20]. Finally, geography, species variation and coastal vs. offshore habitat comparisons were considered potential drivers of the Se/Hg ratio variability affecting the bioavailability and toxicity of MeHg in several fish species of the north-eastern Atlantic [21].

The above considerations suggest that the practice of species substitution includes issues beyond commercial fraud, including considerations related to different levels of bioaccumulation of contaminants among species based on their feeding behavior, trophic level and the habitat occupied, as well as the fishing area of provenience.

For these reasons, in this work two different but complementary approaches [22] were used to evaluate the reliability of fish-based baby food as a source of safe nourishment for babies. The barcoding analysis based on the Cytochrome Oxidase I sequences was be used for fish species authentication and the analysis of metal/metalloid levels was carried out to estimate the exposure risk assessment derived from consumption of selected fish-based baby food in infants and toddlers.

Our final goal is to evaluate if the eventual detection of fish species substitution could affect in some way the fish-based baby food safety in terms of metal/metalloid content.

## 2. Results

The examined samples of fish-based baby foods contain fish fillets, identified by the common name of the species, steamed with potatoes or vegetables. Almost all brands claim to contain 18% of fish and only two brands contain 20% of fish. One brand claimed to contain two species of fish but for this sample DNA extraction failed. Only one brand declared on the label the scientific name of the fish species.

### 2.1. DNA Barcoding

Cytochrome c oxidase I (COI) DNA sequences were obtained from all processed samples except from a fish puree sample containing 9% salmon and 9% hake, for which DNA extraction was unsuccessful. Five DNA samples were extracted for each brand, except for the brands named PL01, PL02, L01 and PP01, for which only one DNA sample was successfully obtained probably due to the bad quality of DNA. Presence of multiple fish species was not detected in the examined products (Table 1). The length range of the 69 obtained COI sequences was between 648 bases and 655 bases due to their different end length. For these sequences no insertions, deletions or stop codons were observed and NUMT were not sequenced [23]. Eleven species were identified in all examined baby food products—*Sparus aurata* (Sparidae), *Pleuronectes platessa* (Pleuronectidae), *Dicentrarchus labrax* (Moronidae), *Salmo trutta* (Salmonidae), *Merluccius merluccius* (Merlucciidae), *Merluccius australis* (Merluccidae), *Salmo salar* (Salmonidae), *Oncorhynchus nerka* (Salmonidae), *Oncorhynchus mykiss* (Salmonidae), *Gadus morhua* (Gadidae) and *Gadus chalcogrammus* (Gadidae). The identity percentage between the COI query sequences and their top-match sequences ranged from 98.92 to 99.69 with 100% of sequence coverage (Table 1).

In three cases, the species detected through COI DNA barcoding did not match with the common name of the species shown on the label. In particular, the rainbow trout, *Oncorhynchus mykiss*, was found instead of the salmon *Salmo salar* and the Alaska pollock, *G. chalcogrammus* and the Southern hake, *M. australis* were found instead of European hake, *M. merluccius*.

### 2.2. Metal/Metalloid Levels and Exposure Risk Assessment

Descriptive statistics of the analyzed elements are shown in Table 2. All the results for Co, Pb, Sb and V were below the LOD. For the other elements, we found the following percentages of values below the LOD—61.1% for Cd; 66.6% for Hg; and 22.2% for Ni. Furthermore, 38.9% of values for Cd and 16.7% of values for Ni were also below the LOQ. For the statistical elaboration, values below the LOD were elaborated as LOD/2.

Table 3 reports the Estimated Daily Intake (EDI) for infants and toddlers. Comparing EDI calculated for toxic metals, we revealed that infant and toddler oral exposure was lower than the provisional tolerable daily intake (PTDI) fixed by the European Safety Authority (EFSA) respectively by—2.26 and 3.06 fold for As; 12.3 and 17 fold for Cd; 2.41 and 3.27 fold for Hg; and 14.7 and 18.9 fold for Ni. Regarding Cr, although in its trivalent form it is considered essential, at present, the mechanism(s) for these roles and the essential function of chromium in metabolism have not been substantiated [24] and a PTDI was established. Accordingly, we found that infant and toddler oral exposure was lower than the PTDI respectively by 147 and 199 fold. Overall, the exposure to toxic metals resulting from the ingestion of the selected baby food is minimal but not negligible and it represent only part of the total diet.

In relation to the essential elements Cu, Mn, Se and Zn, we found that EDI was lower than the Dietary Reference Value (DRV) (Table 2). In particular, our data revealed that infant and toddler oral exposure, derived from the consumption of one daily box of fish/vegetable-based baby food, was lower than DRV respectively by 136 and 321 fold for Cu, 112 and 152 fold for Mn, 32 and 42 fold for Se and 167 and 338 fold for Zn. It indicates a low intake of these essential nutrients, which should be supplemented with other kind of foods.

Table 4 reports the Target Hazard Quotient (THQ) and Cancer Risk (CR) for infants and toddlers. For all metals/metalloids, a THQ value below one was obtained for both for infants and toddlers, indicating a low risk to develop chronic systemic effects, although in infants it was slightly higher than toddlers. Nevertheless, the Total Hazard Index derived from the sum of each THQ remained below 1.

Regarding As CR, the probability of developing cancer is 3 in over 1,000,000 in infants and 6 in over 1,000,000 in toddlers, thus sufficiently below the acceptable lifetime risk (ALR) of 10–5.

## 3. Discussion

The results obtained in this study, using two complementary approaches, allowed us to evaluate the reliability of the fish/vegetable-based baby foods as a source of safe nourishment for babies. In particular, the molecular approach proved to be very useful to validate fish species in seafood products because it allowed the identification of a species by analyzing a small piece of fresh tissue from a precooked or cooked food. Furthermore, the molecular approach based on the COI DNA barcoding has become the more widely used tool for molecular species identification in seafood products because a very high number of COI sequences of identified voucher specimens have been collected in public databases for use for search queries [25,26,27,28,29,30,31,32,33,34,35,36]. However, alongside the many advantages provided by COI DNA barcoding in fish species identification, some limitations have been also highlighted such as the inefficiency in the detection of hybrids [37] due to the introgression resulting from hybridization; the presence of multiple mitochondrial pseudogenes in the nuclear genome that could be interpreted as mitochondrial DNA variants [38] heteroplasmy due to the presence of multiple mtDNA haplotypes in a single organism [39]. In our study, the COI DNA barcoding revealed that in three samples the species detected did not match the common name of the species shown on the label. In particular, *G. chalcogrammus* and *M. australis* were found in place *of M. merluccius* and *O. mykiss* was found in place of *S. salar*. These three samples reported the common name of the fish on the labels, respectively hake and salmon, without indicating the scientific name of the species. In this regard, it should be noted that there are rules currently in place mandating origin labelling and an indication of the scientific name of the fish species on the labels (EU Regulation No 1379/2013) but this is not mandatory but only voluntary, for fish products such as prepared or preserved fish. Based on this regulation, all species of fish that constitute an ingredient in a food product may be designated as ‘fish,’ provided that the name and presentation of the food product does not refer to a specific species. Almost all brands of fish-based baby food we examined declared on the label the common name of the species with the exception of one brand (16% of the samples examined in this study), which reported the scientific name of the fish species. However, based on the Italian ministerial decree (MD 31 January 2008 of the Ministry of Agricultural, Food and Forestry Policies), which indicates the Italian names of the fish species of commercial interest, the common name “hake” must be used only to indicate the European hake *M. merluccius*, while *G. chalcogrammus* must be indicated as Alaska pollock and *M. australis* as Southern hake. In regard to the sample labelled as salmon, the MD establishes that the common name “salmon” must be used to only indicate the species *S. salar*, while *O. mykiss* must be indicated as rainbow trout. Thus, these three samples should be considered mislabeled.

The European hake, *M. merluccius*, is a commercially important fish species appreciated from consumers in southern Europe. Landing of this species in the European states are decreasing [40] and its market value is increasing. For these reasons, species of less commercial value have been found in place of the European hake in processed seafood products [41]. In particular, substitution with other gadoid and other *Merluccius* species are very frequent [42] as we found in our study. The substitution of the marine Atlantic salmon (*S. salar*) with the freshwater rainbow trout (*O. mykiss*) of much lower commercial value is also a well-known commercial fraud driven by the illegal benefits of farmers and merchants worldwide [43,44]. Therefore, our results confirm that species substitution is also practiced in baby products industry. In this case, the geographical origin of the species substituting those declared on the label or the breeding conditions in case of farmed species, could be of concern with respect the level of toxicant bioaccumulation of the species. Indeed, several investigations have detected and statistically assessed intra- and inter-specific variability of metal bioaccumulation in fish species based on their feeding behavior, trophic level and the habitat occupied, as well as the fishing area of provenience [16,17,18,19,20,21,45]. However, in this respect, the results of the second approach based on the exposure risk assessment provided in this study indicate a low risk of developing chronic systemic and carcinogenic effects in infants (6–12 months) and toddlers (1–3 years), under an exposure scenario based on a daily consumption of a single baby food box of fish/vegetable-based food. According to EFSA, the total mean food consumption (g/kg bw per day) for infants (6–12 months) is 106.9 and for toddlers (1–3 years) 114.4 [46]. Consequently, a daily consumption of a single box of fish/vegetable-based baby food (80 g.) represents 8.5% of the total daily diet for an infant of 8.8 kg body weight [46] and 5.9% of the total daily diet for a toddler of 11.9 kg body weight [46]. Thus, it is of fundamental importance that the rest of the daily diet maintain concentrations of toxic metal contents similar to those found in our samples, if not even lower, especially as regards to As and Hg in their inorganic and organic forms respectively, which showed EDI values close to the PTDI.

Oral exposure to inorganic As, has a number of effects, including effects on cardiovascular, respiratory, gastrointestinal, hematological, immune, reproductive and nervous systems [47,48]. Furthermore, the carcinogenic potential in humans is clearly evident [49], including transplacental carcinogenesis [50,51]. The major contributors to the overall oral exposure to total As, originates from all vegetables, nuts and pulses, fruit and vegetable juices, soft drinks and bottled water, coffee, tea and cocoa, alcoholic beverages, miscellaneous food and food for special dietary uses [49]. Nevertheless, drinking water can be a major contributor of inorganic As, in the diet especially in areas with high natural levels [24,52]. Among the solid foods, the predominant intake of inorganic As, is from cereal and cereal products and vegetables, which have been reported to contain on average from 30% to 100% inorganic As [53,54,55] versus a range from 0.14% to 3% in seafood [56]

In addition, methylmercury has a number of health effects such as cardiovascular disease [57], adult and developmental neurotoxicity [58] and developmental immunotoxicity [59]. In the specific case of methylmercury, fish meat is the dominant contributor to dietary exposure for all age classes, followed by fish products [60,61,62,63]. In particular, tuna, swordfish, cod, whiting, pike and hake were major contributors to methylmercury dietary exposure in children [64].

Among essential elements, the contribution to the recommended daily dose derived from a single daily consumption of a fish/vegetable-based food box is extremely low, especially for Cu, Mn and Zn, which is crucial for normal human physiological maintenance [65], at levels between 0.3 and 0.7% of the RDV. Essential micronutrient and mineral deficiencies may induce epigenetic alterations and play a possible role in neurodevelopmental disorders [66]. Thus, it is important, especially for infants and toddlers, to have a proper nutritional intake in the daily diet.

Cu is an essential micronutrient required for lipid metabolism, mitochondrial function, iron metabolism and antioxidant defense [67]. Being a central component of many enzymes, Cu transport systems play an essential role in the physiological responses of cardiovascular cells, including cell growth, migration, angiogenesis and wound repair [68] and in neurotransmitter synthesis, energy metabolism and collagen and elastin cross-linking [24]. A deficit of Cu, as well as iron, is known to lead to impairment of hemoglobin synthesis and consequently reduces the oxygen carrying capacity of erythrocytes, resulting in systemic tissue hypoxia [69]. The main food group contributing to Cu intake for all population groups except infants is grains and grain-based products [24]. Another important contributor to Cu intake is the food group meat and meat products [24].

Manganese is also required for the metabolism of proteins, lipids and carbohydrates and acts as a cofactor for numerous kinases and other enzymes [70]. It is a component of metalloenzymes such as superoxide dismutase, arginase and pyruvate carboxylase [24]. It is an essential micronutrient involved in the normal development of many organs including the brain [71]. A specific Mn deficiency syndrome has not been described in humans [24], although a recent study highlighted that Mn levels measured in hair were inversely correlated to the cognitive level in children of 2–4 years of age [66]. From the diet, the main contributors to Mn intake are cereal-based products, vegetables, fruits and fruit products and beverages [24].

Zinc has been implicated in multiple phases of cellular metabolism and is essential for the action of more than 100 enzymes [72,73]. It has a wide array of vital physiological functions. For example, it is necessary for the functional and structural integrity of cells [74], for the anti-inflammatory response [75] and in bone homeostasis [76]. A deficiency in Zn levels has been associated with autism spectrum disorder (ASD) and in particular with more severe autism symptoms [66]. The main food groups contributing to Zn intake are meat and meat products, grains and grain-based products and milk and dairy products [24].

The most representative micronutrient in the baby foods analyzed is Se, whose absorption contributes to 3.2% of the total RDV in infants and to 2.34% of the same in toddlers. Selenium is an essential micronutrient required for the function of approximately 25 important proteins [77]. It represents an integral part of the enzyme glutathione peroxidase, which has an important antioxidant function including protection of hemoglobin [78]. It plays a crucial role in development and a wide variety of physiological processes including immune responses [79]. It is involved in thyroid hormone synthesis and function [80]. Despite its nutritional benefits, it is one of the most toxic naturally occurring elements and its deficiency and overexposure have been associated with adverse health effects [81]. In the diet, Se is mainly present in organic compounds, such as L-selenomethionine and L-selenocysteine, with lower amounts in inorganic compounds, such as selenate and selenite and it appears to be well absorbed in its various forms from the diet [24]. Because quantification and speciation of Se in foods is complex and because there is considerable variation in the Se content of foods, food composition tables are often inaccurate, resulting in imprecise estimates of selenium intake [24]. Recently, a total diet study was carried out in France to characterize the health risk related to chemical residues in food of infants (children under three years of age) [82]. Based on exposure assessment to polychlorinated dibenzo-p-dioxins and polychlorinated biphenyls (PCBs), it was recommended that exposure to these pollutants be reduced. A screening of commercial baby food (including fish/vegetables-based baby food) was conducted in Spain [83], for determination of antimony, arsenic, bismuth, tellurium (as toxic elements) and selenium (as an essential element). The results indicated a high contribution to the tolerable and recommended infant daily intake of arsenic and selenium from fish-based baby food.

Overall, our results indicates that—(i) while a limited number of brands of fish-based baby food were examined in our study a 18% of species substitution was found, despite the labelling compliance of the products based on current EU regulations; (ii) the exposure to toxic metals resulting from the ingestion of the selected baby food is minimal but not negligible and it represent only part of the total diet; (iii) the selected fish-based baby foods ensure a low intake of essential nutrients, which should be supplemented with other kind of foods; (iv) a total diet evaluation is crucial to balance the negative intake of toxic elements and the positive one of essential nutrients in the baby and toddler diet; (v) all together these results suggest that more attention should be paid by authorities to ensure the safety of food for infants and toddlers.

## 4. Material and Methods

### 4.1. Sampling

The first criterion of sampling of fish based baby food was the screening of commercial brands most commonly sold in Italy and covering the product range in terms of fish species proposed to consumers by each brand. A second criterion was to analyze the products of different brands containing the same fish species. Eighteen fish/vegetable-based baby food samples of eight different brands were then purchased from pharmacies and supermarkets during 2019 and processed for DNA analysis and metal/metalloid extraction and quantification to detect any differences in terms of species authentication and metal/metalloid content.

### 4.2. DNA Analysis

A total of 90 samples were processed for DNA analysis. For each brand 5 DNA extraction were replicated to investigate the presence of multiple fish species in the product. The fish puree samples were centrifuged at 8000 rpm for a minute, at least three times, to remove the liquid components and then preserved in 95% ethanol (J.T. Baker, Deventer, The Netherlands). Total genomic DNA was extracted using the NucleoSpin TM Tissue (Macherey-Nagel) extraction kit following the manufacturer’s protocol and was eluted in 50 µL of molecular biology grade water. DNA extraction success was verified by 1% agarose gel electrophoresis. A portion of about 650 bases of the COI gene was amplified following the PCR conditions reported by Pappalardo et al. [32], in a 20 µL reaction mixture also containing the M13 tailed primers (VF2_t1 and FishR2_t1) described in Ivanova et al. [84] to improve the sequencing quality of the PCR products. Amplicon occurrence was verified by electrophoresis on a 0.8% agarose gel and the PCR products were visualized with SYBR^®^ Safe (Thermo Fisher, Waltham, MA, USA), displayed through a Safe Imager TM 2.0 Blue Light Transilluminator (Thermo Fisher, Waltham, MA, USA). All amplicons were purified using the QIAquick PCR purification kit (Qiagen, Hilden, Germany) and were then bidirectionally sequenced using an ABI 3730 automated sequencing machine at Genechron Biotech Company (http://www.genechron.it/index.php/sanger-sequencing) and the M13 sequencing primers. The sequence chromatograms were checked visually and assembled. Multiple-sequence alignment was carried out using the online version of MAFFT v.7 [85]. Ambiguous sequences were trimmed and primer sequences were cut. The obtained sequences were carefully checked for the presence of nuclear mitochondrial pseudogenes or NUMTs (nuclear mitochondrial DNA sequences), which could be easily coamplified with orthologous mtDNA sequences [23]. The EMBOSS Transeq tool (http://www.ebi.ac.uk/Tools/st/emboss_transeq) was used to translate the nucleotide sequences to amino acids to check for premature stop codons and to verify that the open reading frames were maintained in the protein-coding locus. To confirm the identity of the amplified sequences, we conducted BLAST (Basic Local Alignment Search) searches in GenBank with default parameters (http://www.ncbi.nlm.nih.gov/genbank/) and also used BOLD database (https://www.boldsystems.org/) to validate our sequences (Table 1). All sequences obtained from the present study were published in the National Center for Biotechnology Information database (NCBI) and their GenBank accession numbers are reported in Table 1.

### 4.3. Metal/Metalloid Extraction and Analysis

The metals/metalloids analyzed were the following—arsenic (As), cadmium (Cd), cobalt (Co), chromium (Cr), copper (Cu), mercury (Hg), manganese (Mn), nickel (Ni), lead (Pb), antymonium (Sb), selenium (Se), vanadium (V) and zinc (Zn).

Aliquots of ≈ 0.5 g of wet samples were weighed using an analytical balance (Mettler Toledo) and then mineralized in a microwave oven (Ethos, TC, Milestone), equipped with Teflon vessels, with 6 mL of 67% superpure nitric acid (HNO_3_; Carlo Erba, Italy) and 2 mL of 30% hydrogen peroxide (H_2_O_2_; Carlo Erba, Italy) for 30 min at 80 °C. After acid digestion, the contents of the vessels were decanted into Falcon tubes and double-distilled water (Merck, Kenilworth, NJ, USA) was added to the samples up to 50 mL. A 10 mL rate from each digested sample was taken and 50 µg/L of internal standard (Yttrium, Y and Rhenium, Re, 1000 mg/L Merck, USA) was added to quantify metals with an Inductively Coupled Plasma–Mass Spectrometer (ICP–MS) Elan-DRC-e (Perkin Elmer, Waltham, MA, USA).

Concentrations were determined using standard solutions prepared in the same acid matrix. Standards for the instrument calibration were prepared based on mono element certified reference solution ICP Standard (Merck, USA). For the quality control, a sample for each batch of mineralization was processed in duplicates; one was spiked with a multi-element solution of 25 mg/L with 5 mg/kg and we obtained a mean recovery of 92–115% for all the metals. The limit of detection (LOD) was calculated based on the 40 CFR 136, EPA procedure for digested analytical blanks using the following equation:

LOD = t (df = n − 1, p = 0.99%) × SD,(1)

where t is the one-tailed Student’s t distribution, df is the degrees of freedom, n is the number of blank replicates, p is the probability and SD is the standard deviation. LOD (mg/Kg *w*.*w*.) calculated for the metals/metalloids analyzed are the following—As (0.004), Cd (0.002), Co (0.009), Cr (0.009), Cu (0.01), Hg (0.009), Mn (0.009), Ni (0.002), Pb (0.003), Sb (0.003), Se (0.003), V (0.002) and Zn (0.015).

The limits of quantification (LOQ) were calculated based the following equation:

LOQ = 10 × SD.(2)

### 4.4. Exposure Risk Assessment

An exposure risk assessment derived from consumption of selected fish- and vegetable-based baby food in infants (6–12 months) and toddlers (1–3 years) was conducted for each metal/metalloid detected with levels above the LOD, by providing the Estimated Daily Intake (EDI) (mg/Kg *b*.*w*. per day), the Target Hazard Quotient (THQ) and the Cancer Risk (CR). It was assumed that the toxic inorganic arsenic was 3% of the total [56] and that methylmercury and Cr III were 100% of the total [46,86].

The Estimated Daily Intake (EDI) was calculated according the following equation:

EDI = (C × IR)/BW,(3)

where C is the metal/metalloid level (mg/Kg *w*.*w*.); IR is the ingestion rate (0.080 Kg according to the contents of each baby food box); BW is the body weight, considered as 8.8 Kg for infants (6–12 months) and 11.9 Kg for toddlers (1–3 years) [46]. EDI of each elements without a biological function, such as As, Cd, Cr, Hg and Ni [46,49,86,87,88], was compared with the Provisional Tolerable Daily Intake (PTDI) fixed by the European Food Safety Authority (EFSA), while EDI of essential micronutrients Cu, Mn, Se and Zn [24] was compared with the corresponded Dietary Reference Value (DRV) fixed by EFSA.

The Target Hazard Quotient (THQ) (unitless) was calculated, replacing the US-EPA oral reference dose (RDo—mg/Kg day) with the most up-to-date provisional tolerable daily intake (PTDI) provided by EFSA, converted from µg/Kg bw day to mg/Kg day on the basis of BW of infants and toddlers provided, according the following equation [89]:

THQ = (EF × ED × IR × C)/(PTDI × BW × AT), (4)

where EF is the exposure frequency or number of exposure events per year of exposure (365 days/year); ED is the exposure duration (infants 0.916 years; toddler 3 years); IR is the ingestion rate (the same used for EDI); C is the metal concentration in the baby food (mg/Kg *w*.*w*.); PTDI is the provisional tolerable daily intake (mg/Kg day); BW is the body weight (the same used for EDI); and AT is the averaging time (equal to EF × ED). The target hazard quotient (THQ) indicates the ratio between exposure and the reference dose and calculations were made using the standard assumption for an integrated US-EPA risk analysis. When THQ risk is above 1, systemic effects may occur, meaning that THQ is higher than the reference daily dose.

Lifetime cancer risk (CR) for inorganic As was obtained by using the cancer slope factor (CSF), provided by EPA only for this metal and was calculated according to the following equation [89]:

CR = C × IR × EF× ED × CSF/BW × LT,(5)

where LT is the lifetime (70 years, converted to days); CSF is the cancer slope factor (mg/Kg day) set by US-EPA only for inorganic As. If CR risk is above the acceptable lifetime risk (ALR) of 10^−5^, a value fixed by the US-EPA [90] and applied in this study, it indicates a probability greater than one in 100,000 of an individual developing cancer.

## 5. Conclusions

Although very strict rules govern the production of baby foods, at least in the European Community, our investigation indicates—(i) the need to denote the scientific name of the species on labels of fish-based baby foods, considering the 18% species substitution rate found in this study; (ii) to define the daily consumption of a single box of fish-based baby food, it is of fundamental importance to consider the levels of toxic metals/metalloids that may be consumed with the rest of the daily diet. In this context, while maintaining the levels of toxicants in fish-based baby food below the maximum permissible level for baby consumption, especially in regard to As and Hg in their inorganic and organic forms, respectively, it must be considered that the level of bioaccumulation of contaminants could vary among fish species based on feeding behavior, trophic level, habitat occupied and fishing area of provenience.

## Figures and Tables

**Table 1:** Samples of puree baby food processed for fish species identification. SN: number of replicated samples for each baby food product; FBBF: Fish based baby food; IDS: Identified species by DNA barcoding; CN: Common name of fish species; GB: GenBank Accession N° of obtained sequences; M-GB BLAST: Matched GenBank Accession from BLAST; M-B: Matched BOLD ID; %: percentage identity with 100% coverage. In bold the mislabeled products due to species substitution. The C01, C02 and C03 products declared on the label the scientific name of the species. The symbol/indicates no data due to unsuccessful DNA extraction.

SN	FBBF	IDS	CN *	GB	M-GB BLAST°	M-B	%
1	Sea bream puree 18%	*Sparus aurata*	Sea bream	MT890545	KC501553	DNATR1582-13	99.39
1	Flounder puree 18%	*Pleuronectes platessa*	Flounder	MT890558	JN312173	BNSF088-11	99.08
5	Sea bass puree 18%	*Dicentrarchus labrax*	Sea bass	MT890534	KJ205274	FCFBI131-06	99.54
5	**Hake puree 18%**	***Gadus chalcogrammus***	**Alaska pollock**	**MT890557**	KX119441	GBMIN120739-17	99.38
5	Trout puree 18%	*Salmo trutta*	Trout	MT890542	MG951583	ANGBF41252-19	99.54
5	Sea bream puree 18%	*Sparus aurata*	Sea bream	MT890546	KC501553	DNATR1582-13	99.54
5	Flounder puree 18%	*Pleuronectes platessa*	Flounder	MT890559	KM654278	GBMIN123265-17	99.39
1	Hake puree	*Merluccius merluccius*	Hake	MT890556	MN893171	GBMNB11492-20	99.39
5	Trout puree 18%	*Salmo trutta*	Trout	MT890541	MG951583	ANGBF41252-19	99.07
0	Fish puree (9% salmon, 9% hake)	/	/	/	/	/	/
1	Salmon puree 18%	*Salmo salar*	Salmon	MT890543	KM287091	GERFW519-13	98.92
5	**Hake puree 20%**	***Merluccius australis***	**Southern hake**	**MT890548**	EU074468	FARG173-06	99.69
5	**Salmon puree 18%**	***Oncorhynchus mykiss***	**Rainbow trout**	**MT890550**	MG951597	ANGBF41123-19	99.08
5	Salmon puree 20%	*Salmo salar*	Salmon	MT890544	KM287091	GERFW519-13	99.69
5	*Oncorhynchus nerka* puree 18%	*Oncorhynchus nerka*	Red salmon	MT890549	MG993162	ANGBF53390-19	99.39
5	*Gadus morhua* puree 20%	*Gadus morhua*	Atlantic cod	MT890547	KX267087	ANGBF22055-19	99.23
5	*Pleuronectes platessa* puree 18%	*Pleuronectes platessa*	Flounder	MT890560	KM654277	GBMIN93793-17	99.69
5	Trout puree 20%	*Salmo trutta*	Trout	MT890540	KC501170	DNATR1199-13.COI-5P	99.54

* Italian Ministerial Decree of 31 January 2008, ° BLAST (Basic Local Alignment Search Tool).

**Table 2:** Descriptive statistics of metal/metalloid levels (mg/Kg *w*.*w*. *).

Statistics	As	Cd	Co	Cr	Cu	Hg	Mn	Ni	Pb	Sb	Se	V	Zn
Mean	0.487	0.003	<LOD	0.224	0.324	0.010	0.491	0.022	<LOD	<LOD	0.052	<LOD	1.583
S.D.	0.511	0.002	/	0.025	0.102	0.002	0.167	0.013	/	/	0.015	/	0.359
Median	0.397	<LOD	<LOD	0.222	0.347	0.009	0.442	0.025	<LOD	<LOD	0.055	<LOD	1.538
Minimum	0.039	<LOD	<LOD	0.172	0.120	<LOD	0.206	<LOD	<LOD	<LOD	0.022	<LOD	1.107
Maximum	1.856	0.007	<LOD	0.294	0.509	0.015	0.827	0.042	<LOD	<LOD	0.077	<LOD	2.693

* *w*.*w*.; wet weight.

**Table 3:** Estimated Daily Intake (EDI) (µg/Kg bw day) calculated for Infants (6–12 months) and Toddlers (1–3 years) compared with the Provisional Tolerable Daily Intake (PTDI) for toxic metals and with the Dietary Reference Value (DRV) for essential micro/macronutrients.

Elements	EDI Infants	EDI Toddler	PTDI	DRV	References *
	(6–12 Months)	(1–3 Years)			
As	0.133	0.098	0.3–8 (**)	/	EFSA (2009)
Cd	0.029	0.021	0.357	/	EFSA (2011)
Cr	2.037	1.506	300	/	EFSA (2014)
Cu	2.949	2.181	/	400 (Infants); 700 (Toddler)	EFSA (2017)
Hg	0.095	0.070	0.229	/	EFSA (2012)
Mn	4.463	3.300	/	500	EFSA (2017)
Ni	0.199	0.148	2.8	/	EFSA (2015)
Se	0.476	0.352	/	15	EFSA (2017)
Zn	14.39	10.64	/	2400 (Infants); 3600 (Toddler)	EFSA (2017)

* References are provided for PTDI and DRV values. ** BM: Benchmark dose provided for As in the absence of a PTDI.

**Table 4:** Target Hazard Quotient (THQ) and Cancer Risk (CR) calculated for Infants and Toddlers.

Elements	THQ Infants	THQ Toddler
	(6–12 Months)	(1–3 Years)
As	5.03 × 10^−2^	2.75 × 10^−2^
Cd	9.16 × 10^−3^	5.01 × 10^−3^
Cr	7.72 × 10^−4^	4.22 × 10^−4^
Hg	4.71 × 10^−2^	2.58 × 10^−2^
Ni	8.10 × 10^−3^	4.43 × 10^−3^
Total HI	1.15 × 10^−1^	6.31 × 10^−2^
Elements	CR Infants	CR Toddler
	(6–12 months)	(1–3 years)
As	2.61 × 10^−6^	6.31 × 10^−6^
